# Identifying crucial lncRNAs and mRNAs in hypoxia-induced A549 lung cancer cells and investigating their underlying mechanisms via high-throughput sequencing

**DOI:** 10.1371/journal.pone.0307954

**Published:** 2024-09-05

**Authors:** Lin Lin, Lili Deng, Yongxia Bao

**Affiliations:** 1 Department of Respiratory Medicine, The Second Affiliated Hospital, Harbin Medical University, Harbin, Heilongjiang, People’s Republic of China; 2 Department of Oncology, The Second Affiliated Hospital of Harbin Medical University, Harbin, Heilongjiang, People’s Republic of China; University of Science and Technology Liaoning, CHINA

## Abstract

**Background:**

Rapid proliferation and outgrowth of tumor cells frequently result in localized hypoxia, which has been implicated in the progression of lung cancer. The present study aimed to identify key long non-coding RNAs (lncRNAs) and messenger RNAs (mRNAs) involved in hypoxia-induced A549 lung cancer cells, and to investigate their potential underlying mechanisms of action.

**Methods:**

High-throughput sequencing was utilized to obtain the expression profiles of lncRNA and mRNA in both hypoxia-induced and normoxia A549 lung cancer cells. Subsequently, a bioinformatics analysis was conducted on the differentially expressed molecules, encompassing functional enrichment analysis, protein-protein interaction (PPI) network analysis, and competitive endogenous RNA (ceRNA) analysis. Finally, the alterations in the expression of key lncRNAs and mRNAs were validated using real-time quantitative PCR (qPCR).

**Results:**

In the study, 1155 mRNAs and 215 lncRNAs were identified as differentially expressed between the hypoxia group and the normoxia group. Functional enrichment analysis revealed that the differentially expressed mRNAs were significantly enriched in various pathways, including the p53 signaling pathway, DNA replication, and the cell cycle. Additionally, key lncRNA-miRNA-mRNA relationships, such as RP11-58O9.2-hsa-miR-6749-3p-XRCC2 and SNAP25-AS1-hsa-miR-6749-3p-TENM4, were identified. Notably, the qPCR assay demonstrated that the expression of SNAP25-AS1, RP11-58O9.2, TENM4, and XRCC2 was downregulated in the hypoxia group compared to the normoxia group. Conversely, the expression of LINC01164, VLDLR-AS1, RP11-14I17.2, and CDKN1A was upregulated.

**Conclusion:**

Our findings suggest a potential involvement of SNAP25-AS1, RP11-58O9.2, TENM4, XRCC2, LINC01164, VLDLR-AS1, RP11-14I17.2, and CDKN1A in the development of hypoxia-induced lung cancer. These key lncRNAs and mRNAs exert their functions through diverse mechanisms, including the competitive endogenous RNA (ceRNA) pathway.

## Introduction

Lung cancer, a malignant tumor disease with a high degree of malignancy, has caused a huge health burden and economic pressure worldwide. Its mortality rate is up to 13.5%, making lung cancer one of the most fatal cancer types [1]. Despite significant advances in the research of lung cancer in the medical field, the causes, development mechanisms, and effective treatment methods of lung cancer still remain challenging. Despite significant and groundbreaking progress in the medical field of lung cancer treatment, unfortunately, the five-year survival rate for patients remains unfavorable [2]. The pathogenesis of lung cancer is a complex process involving the combined effects of multiple factors. Smoking, air pollution, and genetic factors are recognized as the main inducers, but these factors alone are not sufficient to fully explain the occurrence of lung cancer. In the development of lung cancer, the uncontrolled proliferation and growth of tumor cells is a prominent feature, which is often accompanied by the formation of a local hypoxic environment. The abnormal and uncontrolled proliferation and excessive growth of tumor cells often significantly and persistently induce hypoxia in local regions [3]. There is ample and compelling evidence indicating a profoundly close association between this hypoxic microenvironment and the progression of cancer [4,5]. The hypoxic environment has profound effects on the biological behavior of tumor cells. It is reported that compared to cancer cells in normal oxygen conditions, cancer cells under hypoxic conditions exhibit a higher survival rate and proliferation potential after treatment, posing a significant challenge to therapeutic efficacy [6]. Hypoxia-inducible factor (HIF), as a pivotal transcriptional regulator under hypoxic conditions, plays a crucial role in the process of tumorigenesis and metastasis, with far-reaching and vital implications [7,8]. The research found that HIF-1α exhibits significant overexpression in various types of solid tumors, yet its expression level is extremely low in normal healthy tissues. More notably, multiple pieces of evidence suggest that the expression level of HIF-1α is significantly correlated with the prognosis of lung cancer patients. This discovery further underscores the crucial role HIF-1α plays in the occurrence and development of lung cancer, and its importance cannot be overlooked [9].

Meanwhile, non-coding RNAs (ncRNAs), a class of RNA molecules that do not directly participate in protein coding, have gradually emerged as significant players in tumor biology research. Among them, long non-coding RNAs (lncRNAs) have garnered significant attention from researchers due to their unique biological functions and regulatory mechanisms. Increasing research evidence indicates that lncRNAs play a crucial role in tumor formation, progression, and metastasis. Through interactions with proteins, DNA, or RNA, they exert fine-tuned regulation on various aspects, including gene expression, chromatin structure, and cell signaling pathways, thus demonstrating a non-negligible influence. In lung cancer, the abnormal expression of long non-coding RNAs (lncRNAs) is closely associated with the malignancy, invasiveness, and metastatic ability of tumors. Dong et al.’s research revealed that the long non-coding RNA DGCR5 plays a significant role in promoting the progression of lung adenocarcinoma (LUAD) by suppressing the expression of hsa-mir-22-3p [10]. Through further in-depth research, it has been clearly demonstrated that the splice isoform of PD-L1 long non-coding RNA significantly contributes to the progression of lung adenocarcinoma by enhancing c-Myc activity [11]. These studies have not only deepened our understanding of the role of long non-coding RNAs (lncRNAs) in tumor regulation, but also provided new strategies and directions for the treatment of lung cancer. Particularly noteworthy is that lncRNAs can function as miRNA “sponges”, known as endogenous competitive RNAs (ceRNAs), competitively binding to the 3’UTR regions of target mRNAs with miRNAs, thus reducing the inhibitory effect of miRNAs on their target mRNAs. This discovery provides a new perspective for us to understand the complex mechanisms of lncRNAs in tumor regulation. However, despite studies exploring the importance of lncRNA-mediated ceRNAs in LUAD, whether cancer cells under hypoxic conditions can influence the occurrence and progression of LUAD through the ceRNA pathway remains a topic worthy of further investigation.

To address this scientific question, we have employed high-throughput sequencing technology in this study to conduct a comprehensive analysis of the lncRNA and mRNA expression profiles in A549 lung cancer cells under hypoxic and normoxic conditions. By comparing the expression differences between the two groups of cells, we have successfully identified a set of lncRNAs and mRNAs with significantly differential expressions. These differentially expressed molecules may reveal the molecular changes in lung cancer cells under hypoxic conditions, providing strong data support for our further exploration of the relationship between hypoxia and the occurrence and development of lung cancer. Subsequently, we utilized bioinformatics methods to conduct in-depth analyses of these differentially expressed molecules. Functional enrichment analysis revealed the enrichment of these differentially expressed molecules in biological functions, helping us to understand their potential roles in lung cancer development. Protein-protein interaction (PPI) network analysis revealed the interaction relationships of these differentially expressed molecules at the protein level, providing clues for our further research on their functions. The ceRNA analysis uncovered the potential regulatory mechanisms of lncRNAs as miRNA "sponges" in lung cancer development. To validate our findings, we employed real-time quantitative PCR (qPCR) technology to confirm the expression changes of key lncRNAs and mRNAs. This validation process not only proved the reliability of our high-throughput sequencing data, but also provided important reference for our subsequent experimental research. In summary, this study has delved into the expression changes and potential mechanisms of lncRNAs and mRNAs in lung cancer cells under hypoxic conditions through high-throughput sequencing and bioinformatics analysis. These findings not only provide new insights into the underlying molecular mechanisms of lung adenocarcinoma but also offer new ideas and strategies for the prevention and treatment of lung cancer. We look forward to future research that can further uncover the complex mechanisms of lncRNAs in tumor regulation, bringing breakthrough progress in the treatment of lung cancer. Meanwhile, we also hope that the high-throughput sequencing data generated in this study can provide valuable reference for the scientific community. The high-throughput sequencing data generated in this study are publicly accessible via the NCBI SRA database with the accession number SRP411127, accessible at the following link:

https://dataview.ncbi.nlm.nih.gov/object/PRJNA906305?reviewer=dm5r2erlkr9hvdu6vpa4jtsolm.

## Materials and methods

### Cell culture and hypoxia treatment

The human pulmonary adenocarcinoma cell line A549 was obtained from the cell bank of the Chinese Academy of Sciences. Cells are cultured in F-12K medium supplemented with 10% fetal bovine serum (FBS) and 1% Penicillin-Streptomycin (PS). Upon arrival, the cells are first allowed to rest overnight in the incubator to facilitate their adherence to the flask walls. The next day, the culture medium is aspirated, and 1 mL of 0.25% trypsin solution is added (sufficient to cover the entire bottom of the flask). The cells are incubated for 5 minutes while monitoring under a microscope. The digestion process is terminated by adding 6 times the volume of the corresponding medium containing 10% FBS and 1% PS. The cells are then resuspended by gentle pipetting. The cells are centrifuged (1000 rpm for 5 minutes), the supernatant is discarded, and fresh medium is added. The cell pellet is resuspended by pipetting to form a suspension of cells. Appropriate cells are then seeded into a new culture flask and incubated in a 5% CO2 incubator. After the cells adhere to the walls, they are treated with 400 μM CoCl2 for 24 hours. Subsequently, the cells are collected for further qPCR analysis.

### RNA extraction

The collected cell samples are mixed with 1 ml of RNAiso Plus, homogenized, and incubated at room temperature for 15 minutes to allow for complete lysis. The lysate is then transferred into a 1.5 ml centrifuge tube. 200 μl of chloroform is added, followed by vortex mixing and incubation at room temperature for 15 minutes. The mixture is centrifuged at 4°C and 12,000 g for 15 minutes. The upper aqueous phase is aspirated into a separate centrifuge tube, taking care not to disturb the interphase. 0.5 ml of isopropanol is added, mixed, and incubated at -20°C to precipitate the RNA. The mixture is centrifuged at 4°C and 12,000 g for 10 minutes. The supernatant is discarded, and the RNA pellet is left at the bottom of the tube. 1 ml of 75% ethanol is added, and the tube is gently vortexed to suspend the pellet. The mixture is centrifuged at 4°C and 12,000 g for 5 minutes, and the supernatant is discarded as much as possible. The RNA pellet is allowed to air-dry or vacuum-dry for 5–10 minutes. The RNA sample is dissolved in 20 μl of DEPC-treated H2O at 55–60°C for 5–10 minutes.

### Measurement of RNA purity and quantification of RNA

Using DEPC-treated H_2_O as a control (Blank), 2 μl of RNA solution is dispensed onto a microplate reader to assess the concentration and quality of the sample.

### RNA reverse transcription

The reverse transcription system consists of 4 μl of 5x primeScript RT Master MIX (perfect Real Time), 0.5 μg of Total RNA, and RNase Free water up to a total volume of 20 μl. The reaction conditions are as follows: incubation at 37°C for 30 minutes, followed by denaturation at 85°C for 5 seconds.

### PCR amplification reaction system

For the qPCR reaction, a total volume of 5 μl SYBR Premix EX Taq (2x) was used, along with 0.3 μl of 10 μM forward primer, 0.3 μl of 10 μM reverse primer, and 3.4 μl of cDNA diluted to a consistent level with water.

### PCR amplification reaction conditions

The qPCR cycling conditions were as follows: initial denaturation at 95.0°C for 3 minutes, followed by 40 cycles of denaturation at 95.0°C for 10 seconds and annealing/extension at 60.0°C for 30 seconds. Prior to these cycles, a pre-incubation step was performed at 50.0°C for 3 minutes. A melt curve analysis was then conducted by gradually increasing the temperature from 60°C to 95°C, with increments of 0.5°C every 10 seconds and continuous plate reading. The sequences of the primers used in this study are detailed in Table 1.

**Table 1:** The names and sequences of all the primers.

primer name	Primer Sequence (5′ > 3′)
GAPDH	F-TGACAACTTTGGTATCGTGGAAGG
	R-AGGCAGGGATGATGTTCTGGAGAG
TENM4	F-CAAAGCCCCGCAGAAATCGTA
	R-AGGCCAATGTCTGTCCGGTA
XRCC2	F-GGGCGATGTGTAGTGCCTT
	R-CTTCTACCTTCAAGTCGGGCA
HDAC5	F-CAGGTCGGGAACCATCCTTG
	R-GGAACTGGGCATGGCTCTT
CDKN1A	F-TGTCCGTCAGAACCCATGC
	R-AAAGTCGAAGTTCCATCGCTC
ARFGEF3	F-GAATGCCGTGAAAGTGACGC
	R-ATGTACGTCTCAATGCACACC
SNAP25-AS1	F-AGGGTTAGAACGACAGGGGA
	R-GACTTCCATGGCTCTCTTTTGA
RP11-58O9.2	F-CAGACCTGCTCCAGACTTCG
	R-TCTCTTGCATCGCTTGCTGA
LINC01164	F-TCCACATCACGTCACCACTG
	R-CAGAAGTCGTCGTTTCCCCA
VLDLR-AS1	F-GCCTTAAAGGATGAGGAGGAGC
	R-CCTTCTGGGCCTAAAGCAGA
RP11-14I17.2	F-AAATCCCATCGCAGAGACAG
	R-TCCCATGTGCTATCTCCAAGT
Hif-1α	F-TATGAGCCAGAAGAACTTTTAGGC
	R-CACCTCTTTTGGCAAGCATCCTG

### Sample information

The six samples include: A549 cells grown under normoxic conditions (k1, k3, k4) and A549 cells treated with hypoxia (c1, c4, c5).

### Construction of the cDNA library

In a rigorous experimental process, we first precisely extracted total RNA from clinical tissue samples using the RNAiso Plus Kit provided by TaKaRa Company, located in Shiga, Japan. Subsequently, to ensure that the purity and concentration of the RNA meet high standards, we conducted thorough testing using agarose gel electrophoresis and the Nanodrop spectrophotometer from Nanodrop Technologies in Wilmington, Delaware, USA. Based on these results, we successfully constructed a poly(A)-tailed enriched cDNA library following industry-recognized standard methods.

### High-throughput sequencing

After RNA extraction, purification, and library preparation for sequencing, the libraries were subjected to paired-end sequencing using Next Generation Sequencing (NGS) based on the Illumina HiSeq 3000 sequencing platform. The statistical power of this experimental design, calculated in RNASeqPower is 0.83. The high-throughput sequencing data generated in this study are publicly accessible via the NCBI SRA database with the accession number SRP411127, accessible at the following link:

https://dataview.ncbi.nlm.nih.gov/object/PRJNA906305?reviewer=dm5r2erlkr9hvdu6vpa4jtsolm.

### Sequencing data quality control

The following quality control steps are performed using the Trimmomatic (v3.6) tool [12]: Reads are trimmed from both ends to remove consecutive bases with a quality score below 10. Reads with fewer than 80% of their bases having a quality score greater than 20 are discarded. Reads with a length shorter than 50 nucleotides are filtered out.

### The clean reads are mapped to the human reference genome

The clean reads are mapped to the human reference genome (GRCh38.p7, GENCODE) using the TopHat2 software (v 2.1.0) [13]. A maximum of four base mismatches are allowed during the alignment, while all other parameters are set to their default values.

### Gene expression quantification

The six samples are re-assembled into transcripts using the StringTie tool (v1.3.3b) [14] referencing the human genome annotation file (GENCODE, v25) [15]. The resulting multiple GTF files are then merged into a more comprehensive transcript annotation. Subsequently, the gene expression levels of individual samples are quantified using the HTSeq tool (V0.6.1p2) [16], yielding read count results. These results are normalized using CPM (counts per millions), and genes with a normalized expression value below 0.1 in fewer than three samples are classified as genes with low expression abundance. The obtained results are further categorized into lncRNA and mRNA based on the annotation information.

### Differential expression analysis

Differential expression analysis is performed using the quasi-likelihood F-tests method implemented in the edgeR software package in R [17]. Significantly differentially expressed lncRNAs and mRNAs between hypoxic and normoxic cells are identified, with genes with low expression abundance removed from the analysis. The threshold is set at |logFC| > 1 and FDR < 0.01. A two-dimensional clustering heatmap and a volcano plot are generated to visualize the differential expression results.

### Gene Ontology (GO) analysis and Kyoto Encyclopedia of Genes and Genomes (KEGG) pathway analysis of differentially expressed mRNAs

The differentially expressed genes (mRNA) are analyzed for their enriched GO functions and KEGG pathways using the DAVID tool (V6.8) [18]. The parameters for enrichment analysis are set as follows: the minimum number of genes for enrichment is count > = 2, and the significance threshold for the hypergeometric test is Pvalue < 0.05 (considered as significant enrichment results).

### Prediction of protein–protein interactions

The differentially expressed genes (mRNA) are analyzed for PPI relationships using the STRING online tool [19]. The Required Confidence (combined score) threshold for PPI is set at >0.7. The Cytoscape [20] tool is employed to construct a protein interaction network and analyze its topological structure. Important nodes in the PPI network are identified based on their ranking in terms of network connectivity. Three network topological properties are utilized to assess the significance of nodes within the network: Degree Centrality, Betweenness Centrality [21], and Closeness Centrality [22]. The CytoNCA [23] plugin for Cytoscape is used for this analysis (parameter setting: network without weight). In the CytoNCA output, higher node scores indicate more significant positions within the network, making them more likely to be key nodes.

### Subnetwork module analysis

The PPI relationship network is analyzed for subnetwork modules using the MCODE tool [24]. Subsequently, the significantly enriched GO functions and KEGG pathways of the module genes are analyzed using the DAVID tool.

### Prediction of lncRNA target genes

For the differentially expressed mRNAs and lncRNAs obtained, the correlation between lncRNAs and mRNAs is calculated using the Spearman rank correlation coefficient. The p-values are corrected using the BH method, and a threshold of p.adj (adjusted p-value) < 0.1 & rho > 0.9 is selected to determine significant correlation between lncRNAs and mRNAs. These mRNAs are considered potential target genes of lncRNAs. The lncRNA-mRNA network is visualized using Cytoscape software.

### Prediction of lncRNA function

The function of lncRNA is predicted through enrichment analysis of GO functions and KEGG pathways for its target genes. This prediction is performed for lncRNA with a target gene count of at least 5. The clusterProfiler [25] package is used to analyze the enrichment of GO functions (Cellular Component (CC), Molecular Function (MF), and Biological Process (BP)) and KEGG pathways for the target genes of each lncRNA. The significance threshold for enrichment is set at p-value < 0.05. To compare the enriched pathways among lncRNA, the top 5 pathways for each lncRNA are presented (selection threshold: p-value < 0.05).

### MiRNA prediction and construction of ceRNA network

The miRNAs regulating the differentially expressed mRNAs are predicted using the TargetScan_microRNA_2017 dataset from the Enrichr tool [26]. The regulation relationships between miRNAs and mRNAs are obtained by selecting results with a p-value < 0.01. For the miRNAs obtained in the previous step, the miRanda tool [27] (parameters: -sc 140, -en -20, -strict) is used to predict the existence of binding sites between miRNAs and lncRNAs, thereby determining potential regulation relationships between miRNAs and lncRNAs. By integrating the miRNA-lncRNA, miRNA-mRNA, and lncRNA-mRNA relationships, a regulatory network of miRNA-lncRNA-mRNA (ceRNA) is constructed. CeRNA refers to mRNAs, lncRNAs, pseudogenes, and other transcripts that can competitively bind to the same miRNAs through microRNA response elements (MREs), regulating each other’s expression levels.

### Statistical analysis

The results are presented in tables as mean ± SEM. Statistical analysis was performed using GraphPad Prism 5 (GraphPad Software, San Diego, CA, USA). The asterisk indicates the significance of the difference: * P<0.05, ** P<0.01, *** P<0.001.

## Results

**Fig 1:**
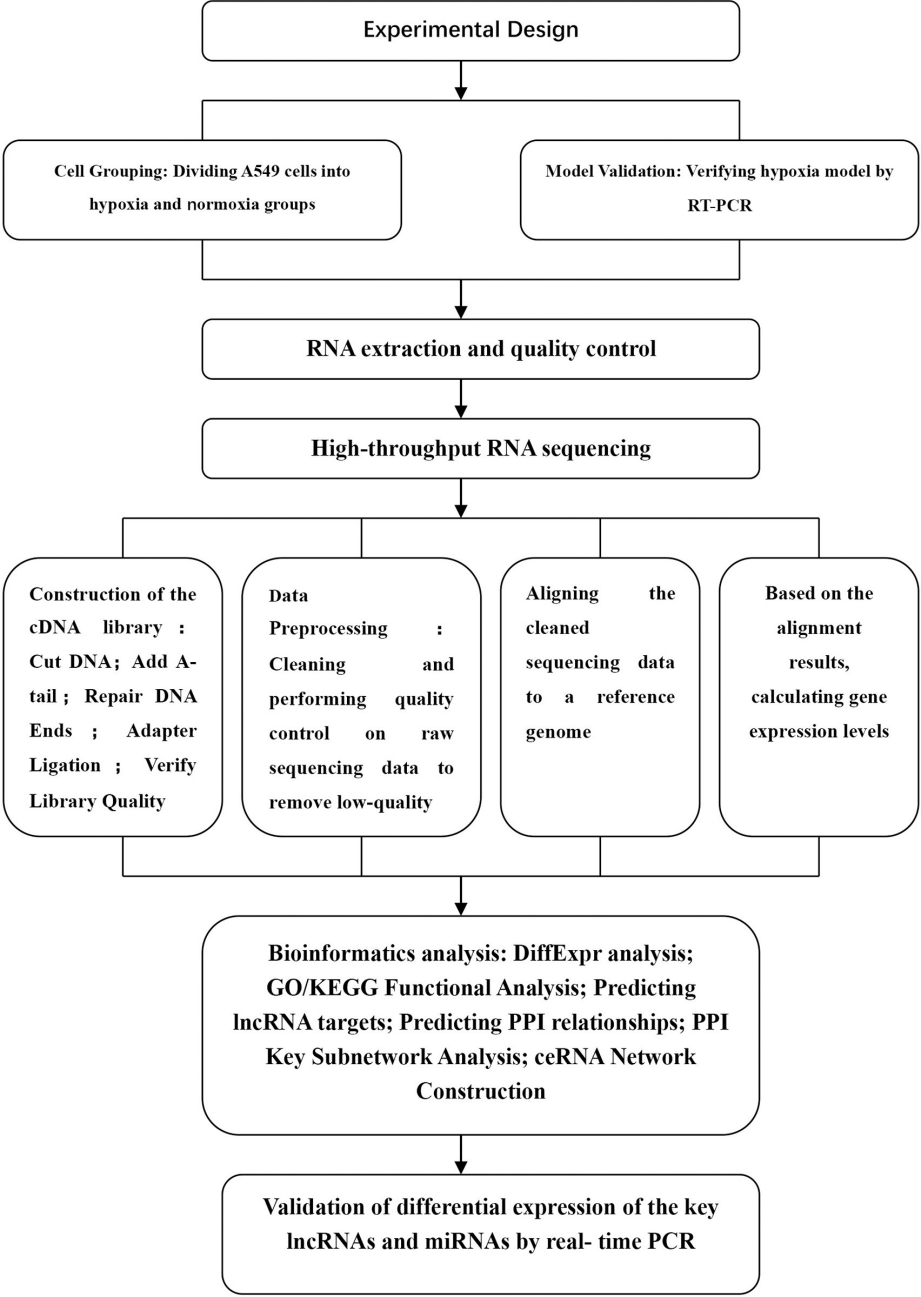
Flowchart of this study.

The statistical power of this experimental design, calculated in RNASeqPower is 0.83. A flowchart for analyses is presented in Fig 1.

### Construction and validation of hypoxic cell model

**Fig 2:**
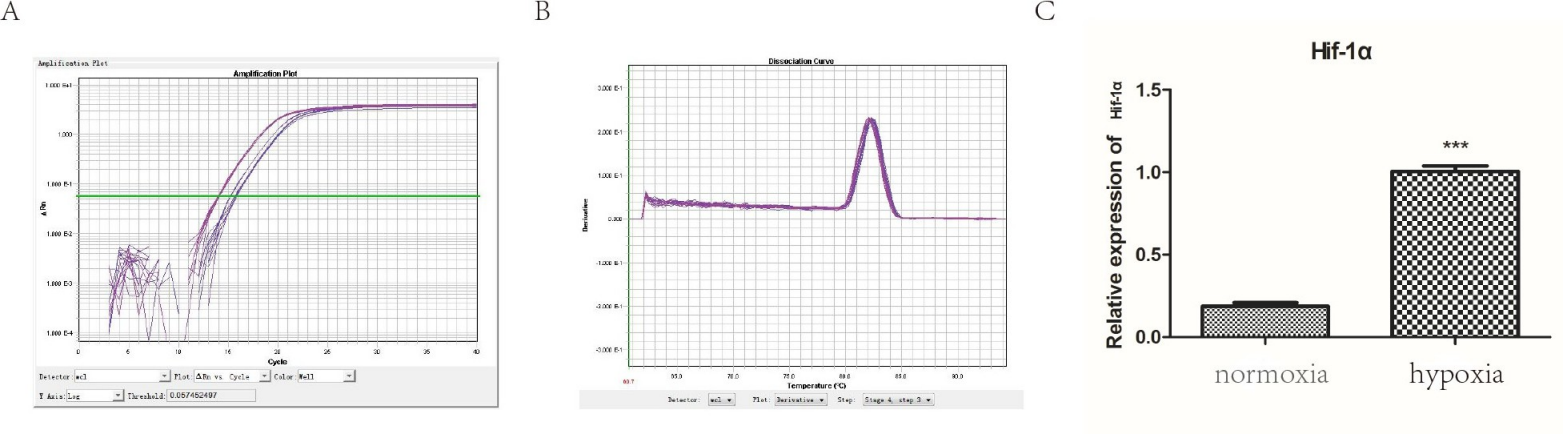
Construction of hypoxia models. (A) Amplification curve. (B) Melting curve. (C) HIF-1α mRNA levels between the two groups.

This is seen in Fig 2, after cells had undergone hypoxia, mRNA levels of HIF-1α in A549 cells were significantly increased (t = 19.94, df = 16, p <0.0001). This showed that our hypoxia model was successful. HIF-1α, hypoxia-inducible factor-1α.

### Results of the sequencing data alignment

In this study, we present the alignment results of sequencing data obtained from different samples. Table 2 summarizes the key metrics, including the total number of input reads, the number of mapped reads, the mapped rate, and the uniquely mapped reads for both left and right paired-end reads. Additionally, the overall read mapping rate is provided for each sample. The results indicate that the sequencing data from different samples have high mapping rates, ranging from 91.00% to 94.67% for left paired-end reads and 91.17% to 93.00% for right paired-end reads. Additionally, the uniquely mapped rates are also relatively high, suggesting the accuracy and reliability of the alignment process. Overall, these results provide a comprehensive overview of the sequencing data alignment and can be used for further bioinformatics analysis and interpretation.

**Table 2:** Alignment results of sequencing data.

Sample	Left Reads Input	Left Reads Mapped Reads	Left Reads Mapped Rate	Left Reads Uniquely Mapped	Right Reads Input	Right Reads Mapped Reads	Right Reads Mapped Rate	Right Reads Uniquely Mapped	Overall Read Mapping Rate
K1	49,398,331	46,765,833	94.67%	41,727,463	49,398,331	45,141,783	91.38%	40,103,413	93.00%
K3	47,768,437	45,141,133	94.50%	40,041,006	47,768,437	43,744,017	91.58%	38,643,890	93.00%
K4	45,012,499	42,471,122	94.35%	37,852,434	45,012,499	41,225,745	91.59%	36,607,057	93.00%
C1	31,090,000	29,173,712	93.84%	26,258,297	31,090,000	28,346,091	91.17%	25,430,676	92.50%
C4	30,564,792	28,472,722	93.16%	25,596,420	30,564,792	27,218,422	89.05%	24,342,120	91.10%
C5	28,433,881	26,425,890	92.94%	23,616,697	28,433,881	25,307,623	89.01%	22,498,430	91.00%

### Differential expression analysis

**Fig 3:**
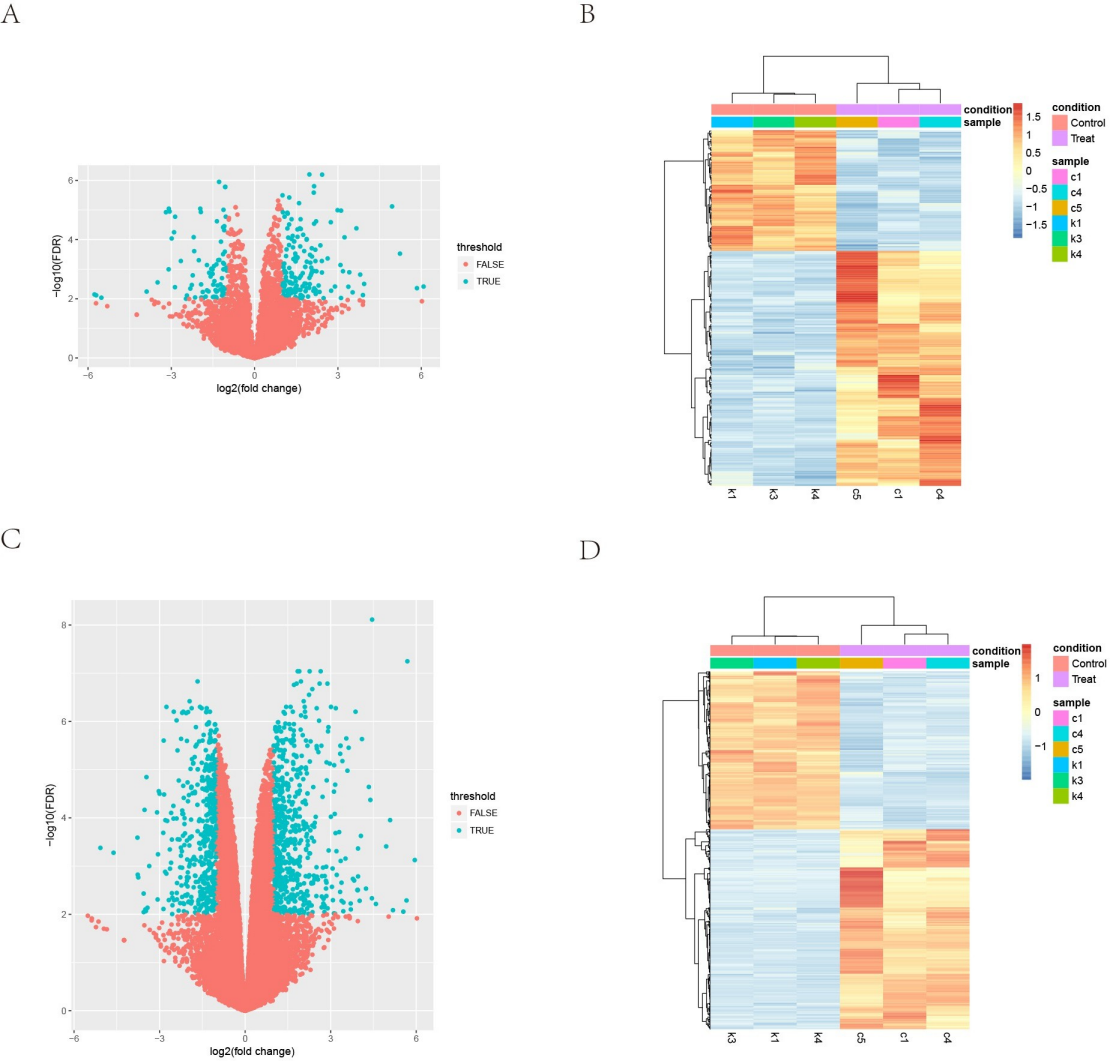
Differential expression analysis. (A) Volcano Plot: Compares lncRNA gene expression between normoxia and hypoxia groups. Each point represents a gene, with log2FC on the x-axis and -log10 (FDR) on the y-axis. Significantly differentially expressed genes are marked with blue dots; others are red. (B) Heat Map: Displays hierarchical clustering of differentially expressed lncRNAs. Yellow indicates high relative expression, while blue represents low relative expression. (C) Volcano Plot: Compares mRNA expression between normoxia and hypoxia groups. (D) Heat Map: Shows hierarchical clustering of differentially expressed mRNAs.

A total of 1370 genes were found to be differentially expressed, including 510 down-regulated mRNAs, 645 up-regulated mRNAs, 73 down-regulated lncRNAs, and 142 up-regulated lncRNAs, based on the criteria of |log2FC| > 1 and FDR < 0.01. Volcano plots and heat maps of differentially expressed mRNAs and lncRNAs are shown in Fig 3.

### GO and KEGG analyses of differentially expressed mRNAs

**Fig 4:**
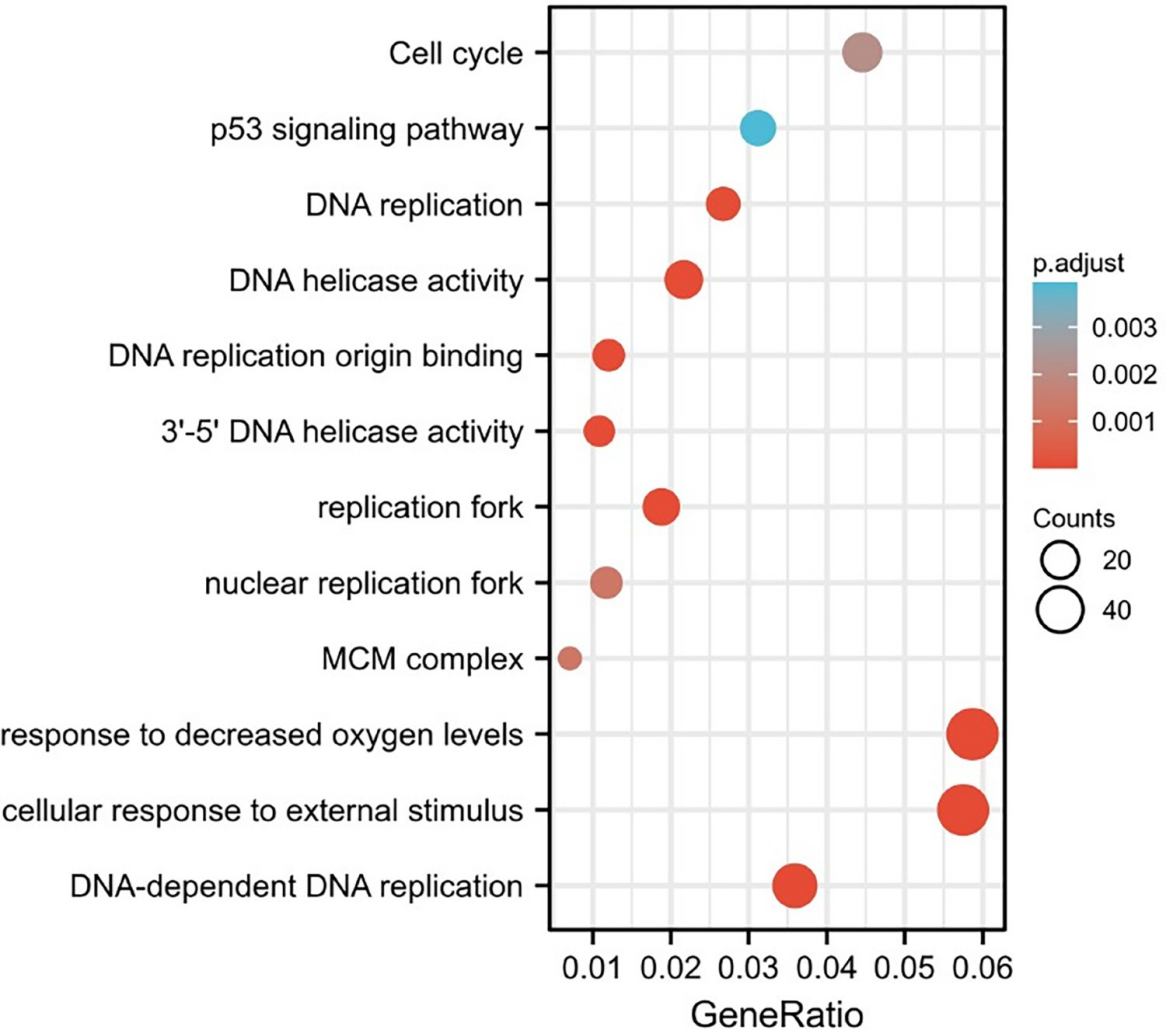
Enrichment analysis of differentially expressed mRNAs. The abscissa represents the number of consensus genes involved in KEGG pathways or GO function annotations. The ordinate represents items of the primary KEGG pathways or GO function annotations.

These differentially expressed genes were mainly enriched in cellular response to external stimulus (BP), DNA-dependent DNA replication (BP), response to decreased oxygen levels (BP), response to oxygen levels (BP), double-strand break repair via break-induced replication (BP), replication fork (CC), DNA helicase activity (MF), DNA replication origin binding (MF), 3’-5’ DNA helicase activity (MF). KEGG pathway analysis showed enrichment of genes associated with p53 signaling pathway, DNA replication and cell cycle et al (Fig 4). See Table 3 for further details.

**Table 3:** Enrichment analysis of differentially expressed mRNAs.

ONTOLOGY	ID	Description	GeneRatio	BgRatio	pvalue	p.adjust	qvalue
BP	GO:0071496	cellular response to external stimulus	48/835	339/18670	1.33e-12	6.95e-09	5.53e-09
BP	GO:0006261	DNA-dependent DNA replication	30/835	153/18670	6.47e-12	1.64e-08	1.30e-08
BP	GO:0036293	response to decreased oxygen levels	49/835	370/18670	9.40e-12	1.64e-08	1.30e-08
BP	GO:0070482	response to oxygen levels	50/835	394/18670	2.79e-11	3.28e-08	2.61e-08
BP	GO:0000727	double-strand break repair via break-induced replication	9/835	11/18670	3.48e-11	3.28e-08	2.61e-08
CC	GO:0005657	replication fork	16/852	70/19717	3.57e-08	1.91e-05	1.86e-05
CC	GO:0042555	MCM complex	6/852	12/19717	4.73e-06	0.001	0.001
CC	GO:0043596	nuclear replication fork	10/852	41/19717	7.12e-06	0.001	0.001
CC	GO:0098687	chromosomal region	29/852	349/19717	6.19e-04	0.083	0.081
MF	GO:0003678	DNA helicase activity	18/831	81/17697	2.87e-08	2.35e-05	2.25e-05
MF	GO:0003688	DNA replication origin binding	10/831	24/17697	5.31e-08	2.35e-05	2.25e-05
MF	GO:0043138	3’-5’ DNA helicase activity	9/831	20/17697	1.12e-07	3.30e-05	3.16e-05
MF	GO:0140097	catalytic activity, acting on DNA	28/831	213/17697	8.33e-07	1.84e-04	1.77e-04
MF	GO:0017116	single-stranded DNA-dependent ATP-dependent DNA helicase activity	8/831	20/17697	1.74e-06	2.57e-04	2.46e-04
KEGG	hsa03030	DNA replication	12/449	36/8076	2.78e-07	8.33e-05	7.78e-05
KEGG	hsa04110	Cell cycle	20/449	124/8076	1.43e-05	0.002	0.002
KEGG	hsa04115	p53 signaling pathway	14/449	73/8076	3.95e-05	0.004	0.004
KEGG	hsa04068	FoxO signaling pathway	17/449	131/8076	8.97e-04	0.063	0.059
KEGG	hsa00010	Glycolysis / Gluconeogenesis	11/449	67/8076	0.001	0.063	0.059

GO: Gene Ontology.

KEGG: Kyoto Encyclopedia of Genes and Genomes.

BP: Biological Process.

CC: Cellular Component.

MF: Molecular Function.

### PPI network diagram of differentially expressed genes

**Fig 5:**
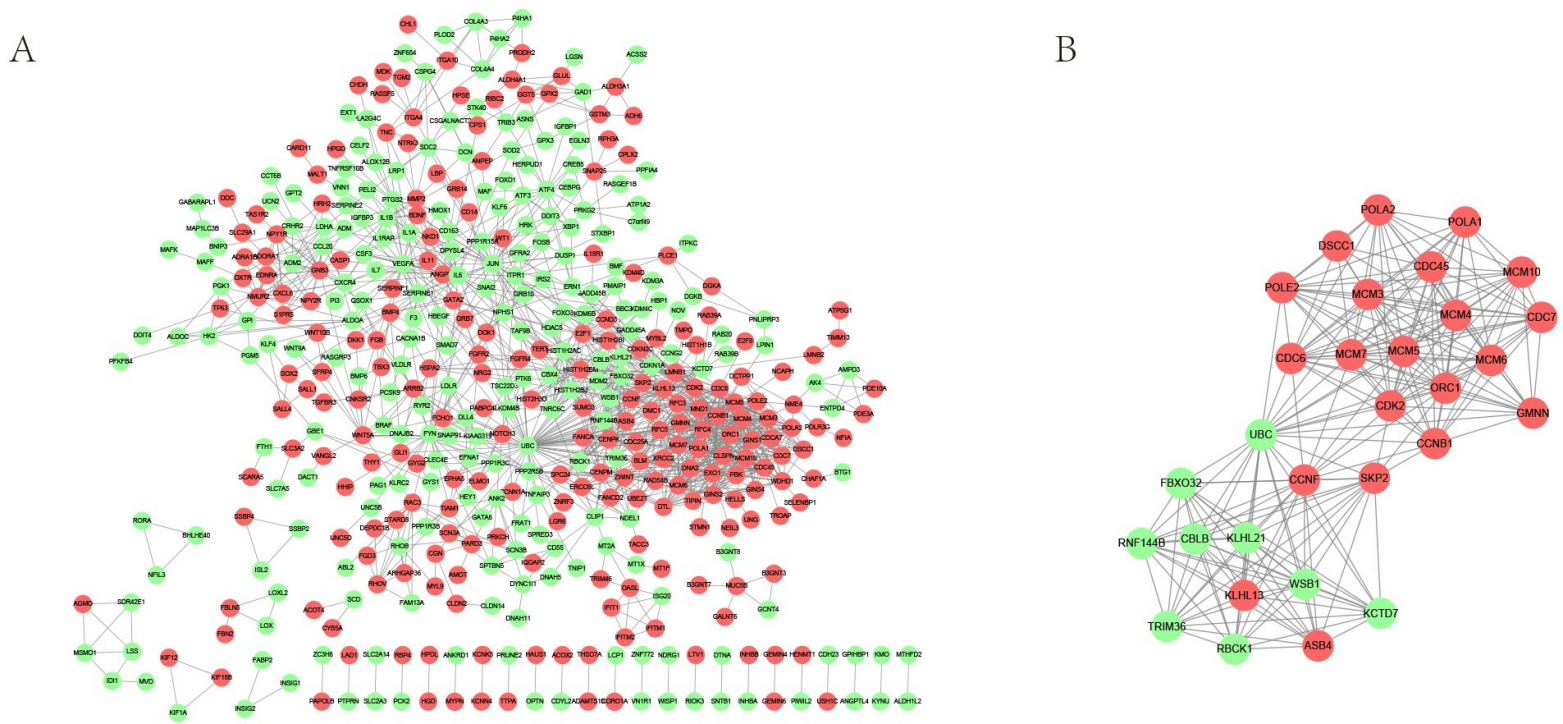
PPI network diagram of differentially expressed genes. (A) Red nodes represent genes with upregulated expression, while green nodes indicate genes with downregulated expression. The network visualizes the interactions among the differentially expressed genes. (B) Subnetwork Modules in Protein-Protein Interaction Networks. In Cytoscape-visualized PPI networks, red nodes signify upregulated genes, while green nodes represent downregulated genes.

The PPI network diagram is shown in Fig 5A, which contains a total of 461 nodes and 1320 interaction pairs. Analysis of key nodes in the network was conducted, and Table 4 lists the top 15 genes with the highest scores for three network topological properties. Among them, four genes—UBC, CCNB1, CDK2, and JUN—are all located within the top 15 scores for all three network topological properties. Using the MCODE plugin in Cytoscape, a subnetwork module of the PPI network was screened with thresholds of score > 10 and node > 10. The resulting subnetwork module is shown in Fig 5B, which contains 30 nodes and 220 interaction pairs.

**Table 4:** Top 15 genes weith highest node connectivity.

Gene	Degree	Betweenness	Closeness
UBC	69	46853.21	0.01260378
CCNB1	44	32057.783	0.01258034
CDK2	36	16684.77	0.01257449
JUN	36	14162.563	0.01256968
RFC4	35	13084.613	0.01256728
CDC45	35	12327.199	0.01256693
MCM4	32	10489.167	0.01256522
CDC6	32	10091.5	0.01256247
MCM10	31	9762.386	0.01254945
MCM7	29	9541.425	0.01254876
MCM3	29	8368.892	0.01254431
POLE2	29	7737.2417	0.01254363
MCM6	28	7105.1797	0.01253679
MCM5	28	6407.623	0.01253509
BLM	27	6137.941	0.01253372

### GO enrichment analysis of subnetwork modules

**Fig 6:**
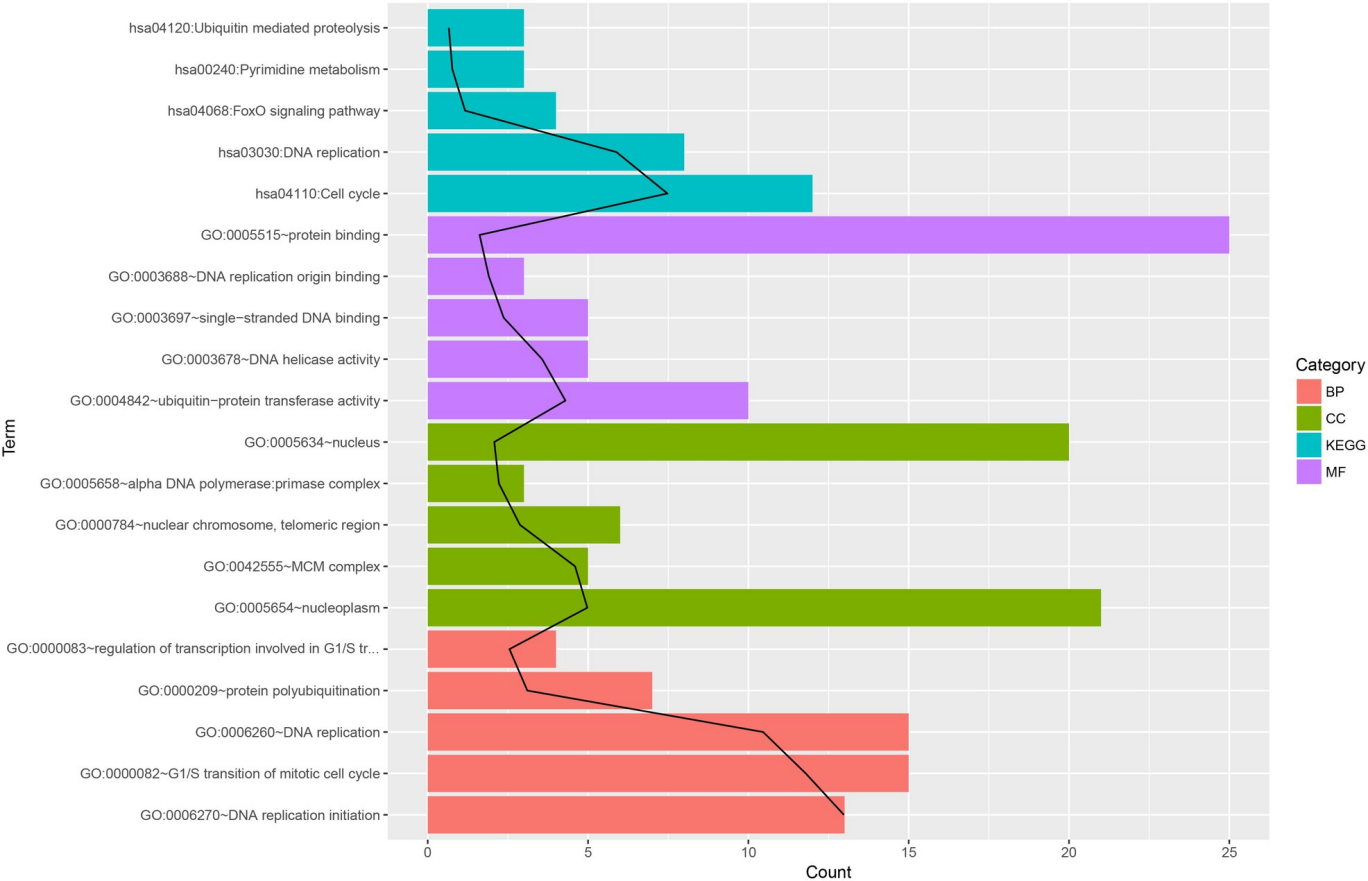
Enrichment analysis of differentially expressed genes (Top 5 by p-value). Category: GO Terms organized into BP (Biological Process), CC (Cellular Component), and MF (Molecular Function). Term: GO function description. Count: Number of differentially expressed genes enriched in each GO Term. Black Trend Line: Represents the significance of enrichment (-log10(p-value)/2).

GO functional enrichment analysis was performed on the subnetwork module, and the results of GO function and KEGG pathway enrichment are presented in Fig 6. Results of functional and pathway enrichment analysis for differentially expressed genes (top 5, sorted by p-value, actual number if less than 5). Category: GO categories, BP refers to biological processes, CC refers to cellular components, and MF refers to molecular functions. Term: GO functional description information. Count: the number of differentially expressed genes enriched in that specific Term. The black trend line represents the value of -log10(p-value)/2.

### Prediction of differentially expressed lncRNA target genes

**Fig 7:**
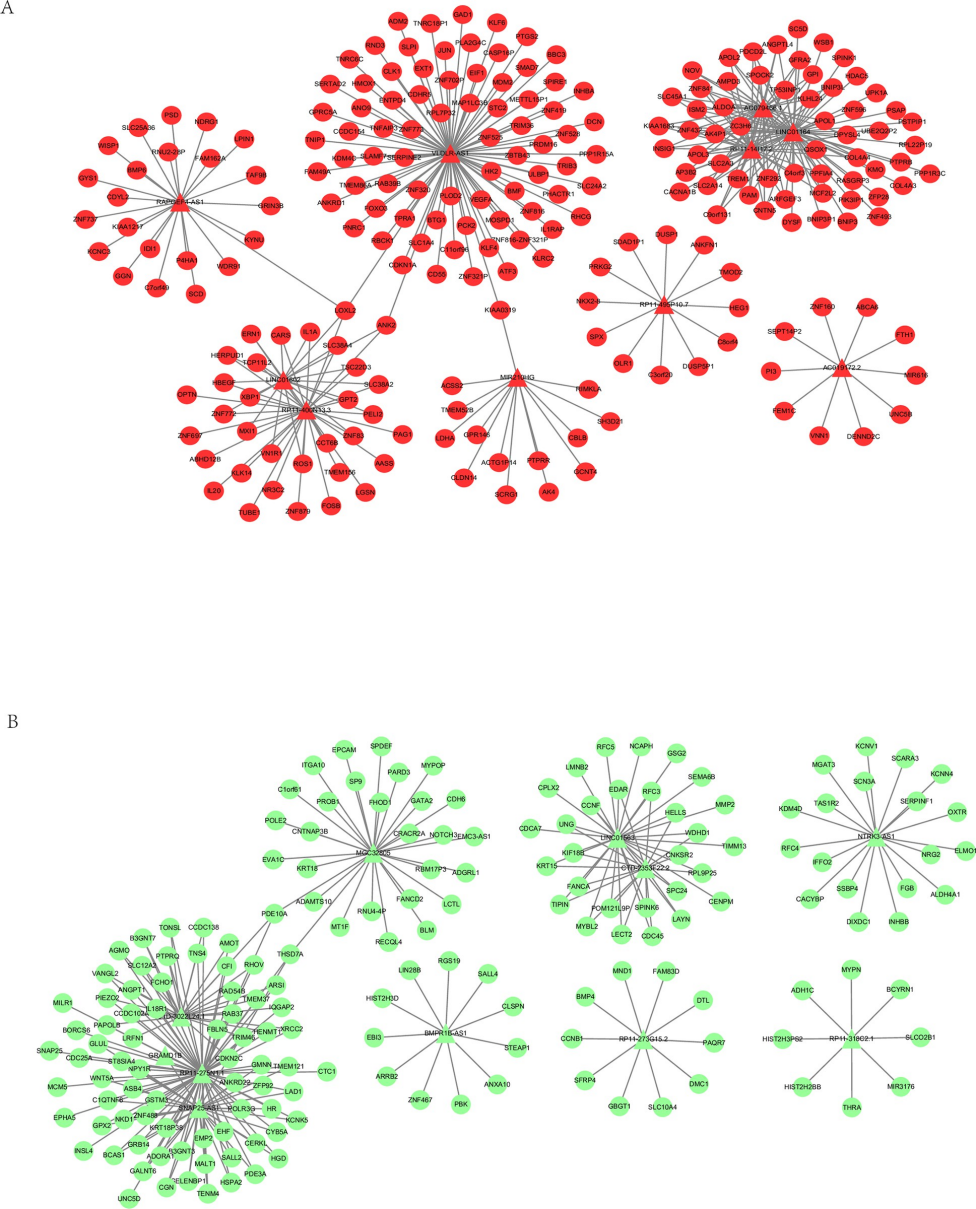
Network graph of differentially expressed lncRNA-Related mRNAs. (A) Red nodes indicate upregulation. (B) Green nodes indicate downregulation. Circular nodes represent mRNAs, while triangular nodes represent lncRNAs.

Using the Spearman rank correlation coefficient, 211 lncRNAs were predicted to have target genes. Analysis was conducted on lncRNAs with five or more target genes. Table 5 lists some of the lncRNAs and their corresponding number of target genes. Fig 7A and 7B depicts the top 10 lncRNA-mRNA network diagrams, each with the largest fold changes (both up and down), which were selected for further analysis. In the lncRNA-mRNA network diagram, red nodes represent up-regulated expression, green nodes represent down-regulated expression, circular nodes represent mRNAs, and triangular nodes with a blue halo represent lncRNAs.

**Table 5:** LncRNA-Target gene counts.

lncRNA	Target_mRNA_num
VLDLR-AS1	95
RP11-275N1.1	82
LINC01164	69
MIR193BHG	59
HOXD-AS2	58
KLHL30-AS1	58
LINC00665	58
RP11-521C22.2	58
C2orf48	45
GACAT2	43

### The prediction for lncRNA function

**Fig 8:**
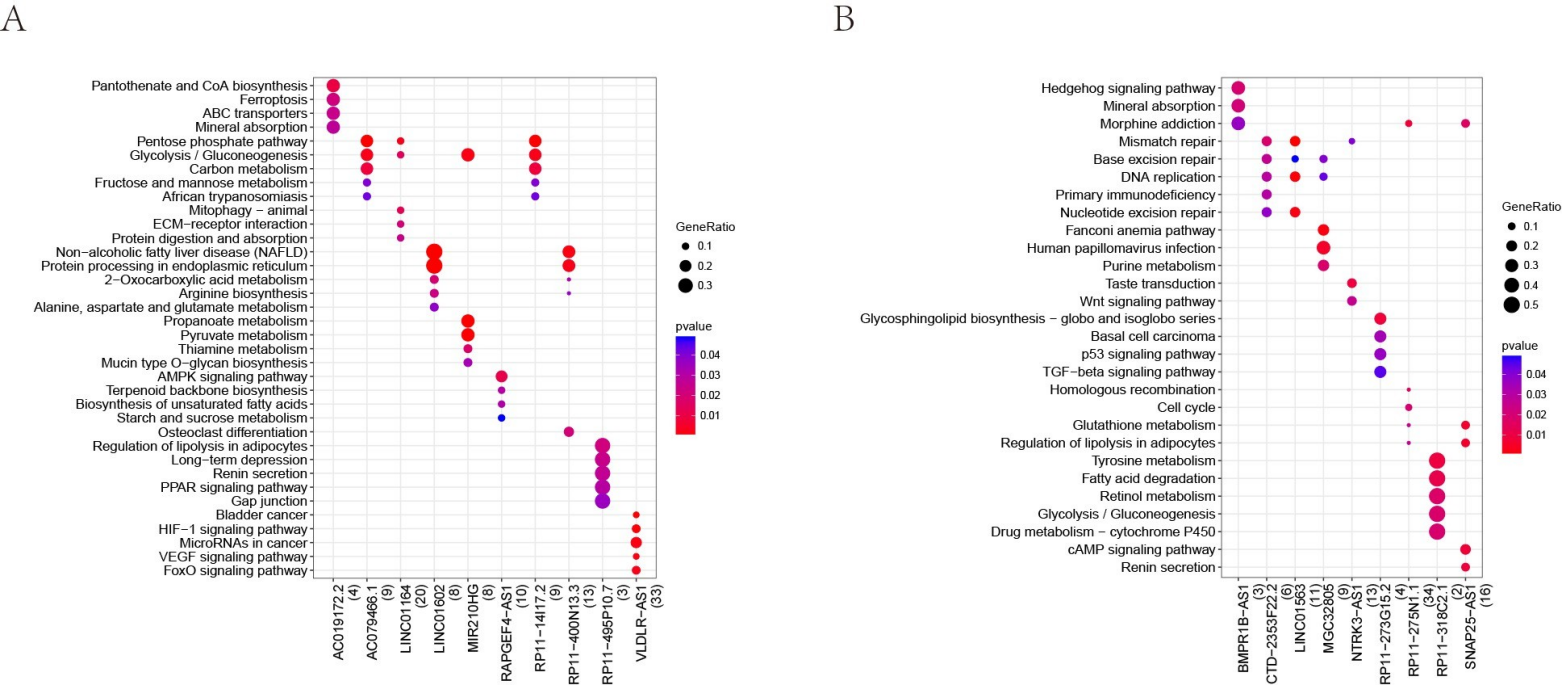
Construction of an lncRNA–miRNA–mRNA ceRNA regulatory network. Triangular nodes denote lncRNAs, blue diamonds indicate miRNAs, round nodes represent mRNAs, arrows indicate regulatory relationships, red color stands for up-regulation, green color represents down-regulation.

We performed GO function and KEGG pathway enrichment analyses for each lncRNA individually. Fig 8A demonstrates the results of a comparative analysis of KEGG pathways impacted by up-regulated lncRNAs. Fig 8B shows the results of a comparative analysis of KEGG pathways impacted by down-regulated lncRNAs. The p-value represents the significance of enrichment in that particular pathway. The GeneRatio refers to the ratio of the number of genes located in the KEGG pathway among the target genes of the given lncRNA to the number of genes annotated in the KEGG database for that pathway. The x-axis represents the lncRNA, and the y-axis represents the name of the enriched pathway.

### The predicted miRNA-mRNA interactions

Table 6 presents a compilation of miRNA predictions made using Enrichr. It includes the miRNA term, the number of mRNAs targeted by each miRNA, the corresponding P-value indicating the statistical significance of the prediction, and a list of the mRNAs that are predicted to be targeted by each miRNA. The miRNAs included in the table are hsa-miR-3150b-3p, hsa-miR-4784, hsa-miR-3145-5p, hsa-miR-1179, and hsa-miR-6882-3p.

**Table 6:** Top 5 miRNA-mRNA prediction results.

Term	mRNA_num	P.value	mRNA
hsa-miR-3150b-3p	28	2.76E-05	KANK2;CDKN1A;SDC2;GATA6;CACNA1B;CBLB;GATA2; NDRG1;EDA2R;LMNB2;BBC3;RNF157;RPH3A;KDELC2;QSOX1; CHAC1;NDEL1;SLC38A4;TRIM67;MPP2;TMEM86A; UNC5B;EIF1;CYP2W1;GPRC5A;PPP1R3B;CDK2;SLC26A9
hsa-miR-4784	28	2.99E-05	KANK2;CDKN1A;SDC2;GATA6;CACNA1B;CBLB;GATA2;NDRG1; EDA2R;LMNB2;BBC3;RNF157;RPH3A;KDELC2;QSOX1;CHAC1; NDEL1;SLC38A4;TRIM67;MPP2;TMEM86A;UNC5B;EIF1; CYP2W1;GPRC5A;PPP1R3B;CDK2;SLC26A9
hsa-miR-3145-5p	13	0.000185	PAQR7;SKIDA1;SERPINE1;TMOD2;LIMD2;CCNB1; NFIA;CBS;AGMO;MDM2;ZNF699;ALDOA;EXPH5
hsa-miR-1179	11	0.000207	CREBRF;SLC2A3;RORA;ALDOA;FOXO3;SOD2;HOXD9; ZWINT;ATF3;LMNB2;CREB5
hsa-miR-6882-3p	12	0.000215	EFNA1;POLA1;SLC7A5;CDKN1A;SCD; DDIT4;KLHL21;PGK1;SLC2A1;AQP3;ZNF488;LMNB2

### Construction ceRNA regulatory network

**Fig 9:**
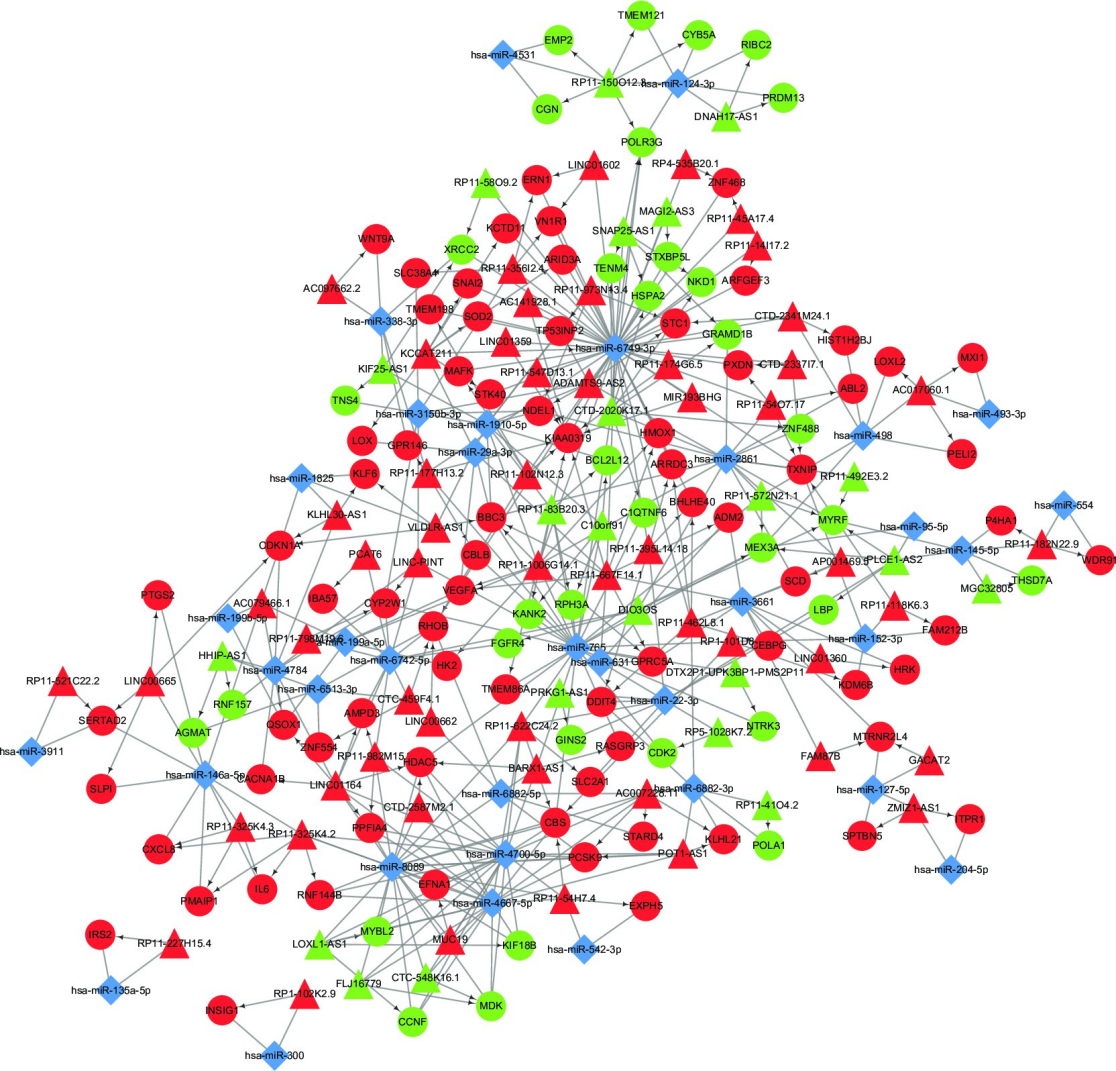
miRNA-lncRNA-mRNA regulatory network diagram. Triangular nodes represent lncRNA, blue diamond nodes represent miRNA, and circular nodes represent mRNA. Arrows indicate the regulatory relationship between lncRNA and mRNA. Red arrows indicate upregulation of expression, while green arrows indicate downregulation of expression.

The binding sites between miRNAs and lncRNAs were predicted using miRanda. By integrating the results of miRNA-lncRNA, miRNA-mRNA, and lncRNA-mRNA interactions, we constructed a miRNA-lncRNA-mRNA regulatory network diagram, as shown in Fig 9. The network diagram demonstrates the interactive relationships among miRNAs, mRNAs, and lncRNAs, with a total of 36 miRNAs, 77 lncRNAs, and 109 mRNAs. Table 7 lists the top 10 lncRNAs with the greatest changes in differential expression in the network, along with their associated miRNAs and mRNAs.

**Table 7:** Information on lncRNAs.

lncRNA	logFC	miRNA	mRNA
SNAP25-AS1	-2.6574	hsa-miR-6749-3p	NKD1,TENM4,POLR3G,GRAMD1B, HSPA2
MGC32805	-2.4712	hsa-miR-145-5p	THSD7A
CTC-548K16.1	-2.22864	hsa-miR-4667-5p,hsa-miR-4700-5p,hsa-miR-8089	MDK
KIF25-AS1	-2.14385	hsa-miR-1910-5p,hsa-miR-338-3p	TNS4,XRCC2
RP11-58O9.2	-1.93679	hsa-miR-6749-3p	XRCC2
LINC01164	5.234304	hsa-miR-4667-5p,hsa-miR-4784,hsa-miR-4700-5p,hsa-miR-6742-5p,hsa-miR-8089	HDAC5,PPFIA4,QSOX1,CACNA1B, ,AMPD3,
AC079466.1	4.949446	hsa-miR-199a-5p,hsa-miR-199b-5p	QSOX1
LINC01602	3.375086	hsa-miR-6749-3p	ERN1,VN1R1
VLDLR-AS1	2.987339	hsa-miR-1910-5p,hsa-miR-1825,hsa-miR-1825	BBC3,CDKN1A,KLF6
RP11-14I17.2	3.113001	hsa-miR-6749-3p	ARFGEF3

### Validation of the differential expression of SNAP25-AS1, RP11-58O9.2, LINC01164, VLDLR-AS1, RP11-14I17.2, TENM4, XRCC2, HDAC5, CDKN1A and ARFGEF3 by real-time PCR

**Fig 10:**
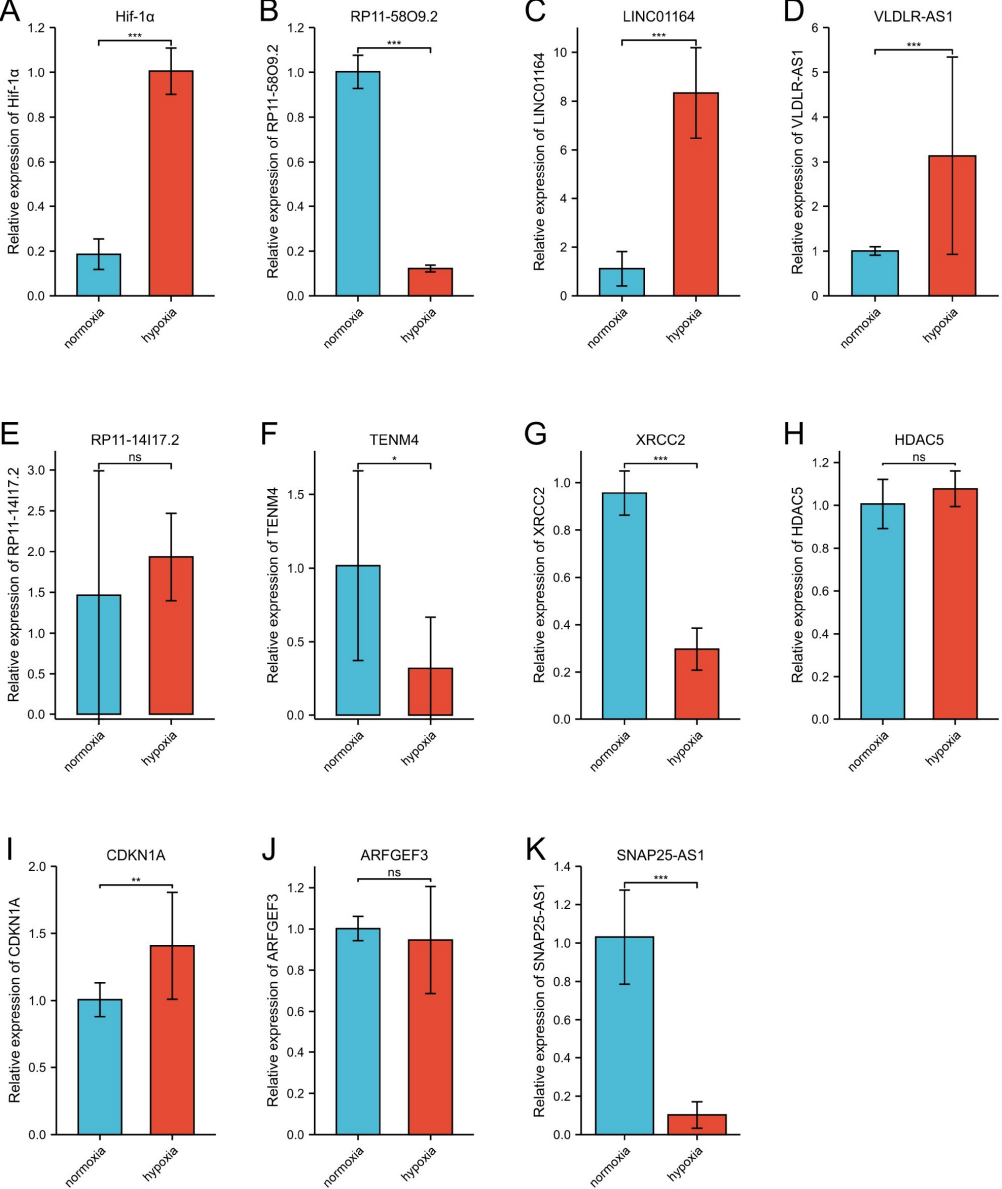
Validation of key gene expressions by qPCR. (A) Expression profile of HIF-1α. (B) Expression profile of SNAP25-AS1. (C) Expression profile of RP11-58O9.2. (D) Expression profile of LINC01164. (E) Expression profile of VLDLR-AS1. (F) Expression profile of RP11-14I17.2. (G) Expression profile of TENM4. (H) Expression profile of XRCC2. (I) Expression profile of HDAC5. (J) Expression profile of CDKN1A. (K) Expression profile of ARFGEF3.

As shown in Fig 10. (A) The difference of Hif-1α expression level is statistically significant between the hypoxic and normoxic groups (t = 19.94, df = 16, p < 0.0001). (B) The difference of RP11-58O9.2 expression level is statistically significant between the hypoxic and normoxic groups (t = 34.9, df = 16, p < 0.0001). (C) The difference of LINC01164 expression level is statistically significant between the hypoxic and normoxic groups (t = 10.93, df = 16, p < 0.0001). (D) The difference of VLDLR-AS1expression level is statistically significant between the hypoxic and normoxic groups (t = 2.896, df = 16, p = 0.01015). (E) The difference of RP11-14I17.2 expression level is not statistically significant between the hypoxic and normoxic groups (t = 0.8679, df = 15, p = 0.3991). (F) The difference of TENM4 expression level is statistically significant between the hypoxic and normoxic groups (t = 3.015, df = 13, p = 0.0099). (G) The difference of XRCC2 expression level is statistically significant between the hypoxic and normoxic groups (t = 14.48, df = 16, p < 0.0001). (H) The difference of HDAC5 expression level is not statistically significant between the hypoxic and normoxic groups (t = 1.504, df = 16, p = 0.1520). (I) The difference of CDKN1A expression level is statistically significant between the hypoxic and normoxic groups (t = 2.881, df = 16, p = 0.0109). (J) The difference of ARFGEF3 expression level is not statistically significant between the hypoxic and normoxic groups (t = 0.6248, df = 1, p = 0.5415). (K) The difference of SNAP25-AS1 expression level is statistically significant between the hypoxic and normoxic groups (t = 0.6248, df = 1, p = 0.5415). The above results show that SNAP25-AS1, RP11-58O9.2, TENM4 and XRCC2 were down-regulated in hypoxia group than that of normoxia group, whereas LINC01164, VLDLR-AS1, RP11-14I17.2 and CDKN1A were up-regulated in hypoxia group. No other between-group differences were statistically significant. See S1 and S2 Figs for dissociation curve and amplification curve. See the S1 Table for raw data from the qRT-PCR experiments.

## Discussion

Under hypoxic conditions, tumor cells activate the HIF signaling pathway, which not only accelerates tumor growth and enhances invasiveness but also promotes tumor metastasis. Despite our current understanding of the relationship between hypoxia and cancer, the molecular mechanisms underlying hypoxia-induced tumorigenesis remain incompletely understood. Exploring and refining these molecular mechanisms could provide novel therapeutic and preventive strategies for lung cancer. In this study, the differentially expressed lncRNAs and mRNAs in hypoxic-induced A549 lung cancer cells were screened by high-throughput sequencing, and the possible mechanism of action was explored. Finally, we identified 1155 mRNAs and 215 lncRNAs differentially expressed in hypoxia vs. control groups. Functional enrichment analysis revealed enrichment in p53 signaling, DNA replication, and cell cycle pathways. Key lncRNA-miRNA-mRNA relationships, including RP11-58O9.2-hsa-miR-6749-3p-XRCC2 and SNAP25-AS1-hsa-miR-6749-3p-TENM4, were identified. qPCR assay confirmed downregulation of SNAP25-AS1, RP11-58O9.2, TENM4, and XRCC2 in hypoxia vs. normoxia, while LINC01164, VLDLR-AS1, RP11-14I17.2, and CDKN1A were upregulated. Our research provides a theoretical reference for improving the understanding of the molecular mechanisms of hypoxia in lung cancer.

Based on the findings of this bioinformatics study, our research team has experimentally demonstrated that RP11-58O9.2 is highly expressed in non-small cell lung cancer cell lines and tissues, and is associated with advanced stage, lymphatic metastasis, and differentiation status. Elevated levels of RP11-58O9.2 are also correlated with shorter survival. Knockdown of RP11-58O9.2 inhibits the proliferation, invasion, and migration of lung cancer cells, as well as tumor growth in mouse xenografts. Furthermore, RP11-58O9.2 may target and regulate miR-6749-3p [28]. These findings further validate the reliability of our research results. In addition, in lung adenocarcinoma, SNAP25-AS1 and MGC32805 have been shown to have prognostic value [29,30]. In addition, previous study indicated that the XRCC2 gene showed more than five-fold higher expression in the patients with lung adenocarcinomas, compared to the control group [31]. The variants rs25487 (XRCC1), rs3218556 (XRCC2), and rs13181 (XPD) all contribute to the efficacy and toxicity of radiotherapy in patients with NSCLC [32]. There is growing evidence that there was higher HDAC5 expression in NSCLC cell lines A549 and CALU-1 compared with BEAS-2B. Fentanyl inhibits lung cancer viability and invasion via repression of HDAC5 CDKN1A upregulation and cisplatin‑pemetrexed resistance in non‑small cell lung cancer cells [33]. In addition, LncRNA AB073614 promotes tumor migration and invasion by repressing CDKN1A in non-small cell lung cancer [34]. Specifically, acting as tumor suppressors or oncogenes, these molecules affect the initiation, development, prognosis and drug-resistance of lung cancer. Interestingly, in our study, these molecules were validated to be differentially expressed in hypoxic lung cancer cells, compared with normoxic cells. In summary, our study demonstrates novelty and importance in multiple aspects compared to established baselines in the field. First and foremost, we have revealed novel associations between lncRNA RP11-58O9.2, miRNA hsa-miR-6749-3p, and mRNA XRCC2, as well as between lncRNA SNAP25-AS1, miRNA hsa-miR-6749-3p, and mRNA TENM4. These interactions, which have been scarcely reported in previous studies, provide a fresh perspective on the complex molecular network of lung cancer. Specifically, the regulatory roles of these lncRNAs and miRNAs offer new insights into the molecular mechanisms of lung cancer and may suggest potential new therapeutic targets. Secondly, we observed significant changes in the expression of specific RNAs under hypoxic conditions. This finding not only sheds light on how hypoxia affects the behavior of lung cancer cells but also aligns with the known metabolic and growth changes in tumor cells under hypoxic stress, further confirming the crucial role of hypoxia in lung cancer development. Moreover, our study revealed up- or downregulation of certain RNAs under hypoxia, providing new clues to understanding the adaptive responses of lung cancer cells to hypoxic environments. Furthermore, for another paper, our research has yielded some valuable discoveries and insights. Firstly, we have confirmed that lncRNA RP11-58O9.2 is significantly overexpressed in non-small cell lung cancer (NSCLC) cell lines and tissues, and this overexpression is closely associated with advanced cancer stages, lymph node metastasis, and the differentiation status of tumor cells. More importantly, we observed a significant correlation between the high expression of RP11-58O9.2 and shorter survival periods among patients, making RP11-58O9.2 a potential biomarker for lung cancer prognosis. Our experiments revealed that when the expression of RP11-58O9.2 is reduced, the proliferation, invasion, and migration abilities of lung cancer cells are significantly inhibited, further emphasizing the crucial role of RP11-58O9.2 in the development of lung cancer [28]. Additionally, this also validates the reliability and effectiveness of the molecular marker identified through our bioinformatics approach in this study. Lastly, functional enrichment analysis identified p53 signaling pathway, DNA replication, and cell cycle pathways as key players in lung cancer. The enrichment of these pathways not only reveals the molecular mechanisms underlying lung cancer development and progression but also aligns with established molecular characteristics of lung cancer, further validating the reliability of our findings.

Despite this, there are still some limitations in our work. First, our research has not yet fully integrated research achievements and technological innovations from multiple fields. Secondly, we have not yet integrated the latest computational biology models into our research methods, which may lead to more innovative discoveries. Therefore, we look forward to future research applying these cutting-edge technologies to clinical practice, thus bringing greater hope to patients. At the same time, we plan to adopt more advanced and efficient data analysis methods in the future in order to achieve more innovative molecular discoveries. Finally, we have only validated some molecules, such as RP11-58O9.2, in the laboratory, and in the future, we will expand these validation methods to other mentioned genes to ensure the accuracy and reliability of the research conclusions. Based on the findings and limitations of the current study, we propose possible directions for future research aimed at further refining and perfecting our discoveries. We recognize that interdisciplinary collaboration and technological integration are crucial for advancing the field of lung cancer treatment. Researchers have successfully developed a highly efficient and outstanding novel circulating tumor cell (CTC) enrichment and separation device, which exhibits remarkable sensitivity in the detection of minimal residual disease (MRD), while possessing broad applicability and a simplified operational workflow [35]. Looking ahead, we will fully explore the practical application potential of this device in the field of lung cancer MRD detection. In addition, cancer stem cells (CSCs) play a pivotal role in post-radiotherapy recurrence, and their strong resistance to radiotherapy may be related to high levels of lysosomes and autophagy [36]. To reduce the risk of recurrence, we will delve deeper into the role of CSCs in lung cancer in the future, aiming to discover more effective therapeutic strategies. It is known that CSCs play a crucial role in tumor recurrence and drug resistance after radiotherapy, as they are able to protect NSCCs from DNA damage caused by radiation, which involves the participation of nitrogen oxides [37]. We hope that through this research, we can develop more effective precision treatment methods for lung cancer. This study has revealed changes in the expression of specific RNA molecules in lung cancer cells under hypoxic conditions, which may be associated with the development of lung cancer. These findings not only help us understand the molecular mechanisms of lung cancer, but also provide new directions for the research and treatment of lung cancer. In the future, we expect to integrate these discoveries with the detection of circulating tumor cells (CTCs) to improve the diagnosis and treatment of lung cancer. However, further exploration of the specific roles of these molecules and considerations on how to apply these findings to clinical practice are still required [38]. In addition, the behavior of cells in a high-salt environment has also attracted our attention. Studies have shown that even if cells are not directly exposed to radiation, a high-salt environment may also increase their risk of damage through a “bystander effect” [39]. This provides us with new therapeutic ideas. We will further explore the specific mechanisms of the effects of high-salt environments on lung cancer cells, including their impacts on cell growth, migration, and RNA expression. Scientists have utilized cancer stem cell (CSC) models and stochastic models to analyze the properties and changes of tumor cells. These models have revealed how NSCC randomly transform into CSC, and predicted the changes in these cells after radiotherapy [40]. Based on experimental data, we have deepened our understanding of the complexity of tumors and revealed the changes of CSCs during radiotherapy. In the future, we will combine models with high-throughput sequencing technology to delve deeper into the molecular mechanisms of key RNAs in lung cancer cells, aiming to better understand the heterogeneity of lung cancer cells and the role of CSCs. FLASH radiotherapy, as a special high-dose radiotherapy technique, aims to effectively kill tumor cells while protecting normal tissues. Research has found that mouse embryonic fibroblasts under hypoxic conditions exhibit stronger radioresistance to FLASH radiotherapy, and their impaired mitochondrial function also enhances this resistance [41]. We plan to continue exploring the impact of FLASH radiotherapy on lung cancer cells and studying the regulatory role of key RNA molecules in response to FLASH radiotherapy, in order to provide new options for lung cancer treatment. Furthermore, with the rapid development of bioinformatics and computational biology, scientists have developed innovative tools such as GFPA, which have greatly propelled research in the field of biology. The GFPA tool has uncovered a close link between genetic variations in CD4 T cells and the CD99 protein, offering a new perspective on understanding their functions and mechanisms. Now, the GFPA tool is publicly available, allowing anyone to utilize it for studying gene-protein relationships. Our research will leverage the GFPA tool to delve deeper into the key RNA molecules in hypoxia-induced A549 lung cancer cells, aiming to gain a better understanding of their functions and interactions with proteins [42]. Moreover, it is well known that long non-coding RNAs (lncRNAs) and microRNAs (miRNAs) play significant roles in cells, but discovering their interactions is time-consuming and laborious. To address this, scientists have proposed the GCNCRF method, which combines Graph Convolutional Neural Networks (GCN) and Conditional Random Fields (CRF), to accurately predict the interactions between lncRNAs and miRNAs by constructing heterogeneous networks. In the future, we will collect relevant RNA data from hypoxia-induced A549 lung cancer cells and employ the GCNCRF method to investigate the interactive network of lncRNAs and miRNAs, aiming to uncover how they impact lung cancer cell growth, metastasis, and drug resistance. This will enhance our understanding of the molecular mechanisms of lung cancer cells under hypoxic conditions and provide novel insights and targets for lung cancer treatment and prevention [43]. In addition, the NDALMA model, which combines lncRNA and miRNA similarity networks with Gaussian Interaction Profile (GIP) kernel similarity, can effectively predict potential associations between lncRNAs and miRNAs. This model has demonstrated good prediction performance in other studies and has been successfully applied to predict lncRNA-miRNA interactions in various human diseases. The datasets and source code used in the research are available at https://github.com/Liu-Lab-Lnu/NDALMA. We plan to incorporate the Network Distance Analysis Model (NDALMA) in our future work to predict lncRNA-miRNA interactions [44]. Metabolism is an important process of material renewal in organisms. Recently, a deep learning algorithm called Graph Convolutional Network combined with Graph Attention Network (GCNAT) has achieved significant results in predicting associations between metabolites and diseases. This algorithm can effectively learn and predict potential associations between RNA molecules and diseases by constructing heterogeneous networks and combining graph convolutional neural networks with graph attention mechanisms. We expect to combine the GCNAT algorithm with high-throughput sequencing technology in future research, bringing more breakthroughs and progress to the field of lung cancer research and treatment [45]. Recently, the DMFGAM deep learning model has achieved significant results in predicting cardiac toxicity related to hERG channel blockers. We plan to utilize this model or other similar techniques to predict the potential cardiac toxicity of key RNA molecules in A549 lung cancer cells under hypoxic conditions. By building a network with graph attention mechanisms, we can gain a better understanding of the roles of these RNA molecules in lung cancer and heart disease, providing new strategies for diagnosis and treatment [46]. Recently, a research study reported a deep learning model called DCAMCP, which is based on a capsule network and attention mechanism, and is capable of accurately predicting the carcinogenicity of compounds. By fusing molecular fingerprint and molecular graph features, DCAMCP has achieved remarkable results in predicting the carcinogenicity of compounds, providing new insights for drug safety evaluation. We plan to combine the DCAMCP model with high-throughput sequencing data to more precisely identify carcinogenic lncRNAs and mRNAs in hypoxia-induced A549 lung cancer cells. First, we can convert the expression profiles of lncRNAs and mRNAs from sequencing data into molecular fingerprint or molecular graph features as inputs for the DCAMCP model [47]. Then, we utilize the DCAMCP model to predict the carcinogenic potential of these RNA molecules and screen out molecules with high carcinogenic risks. The DCAMCP deep learning model developed by scientists, after being trained and validated with extensive drug data, is capable of accurately predicting the carcinogenicity of drugs. The newly proposed single-cell RNA sequencing data analysis framework, scAAGA, has verified its accuracy in COVID-19 blood cell data [48]. In the future, we hope to utilize scAAGA to analyze single-cell RNA sequencing data of lung cancer, aiming to gain a deeper understanding of the heterogeneity and molecular mechanisms of lung cancer cells, and provide effective strategies for precision therapy of lung cancer. In recent years, the interaction between lncRNA and proteins has attracted much attention, but traditional experimental methods have limitations. The LPICGAE deep learning model provides a new method for predicting the relationship between lncRNA and proteins, efficiently predicting potential relationships through autoencoders and matrix reconstruction. Our bioinformatics analysis identified key lncRNA-miRNA-mRNA relationships, which are consistent with the research results of the LPICGAE model, emphasizing the important role of lncRNA in lung cancer [49]. We look forward to future research continuing to explore the relationship between lncRNA and proteins, providing more effective strategies for the treatment of lung cancer and other diseases. Although traditional experiments provide valuable insights into the relationship between metabolites and diseases, they are cumbersome and time-consuming. The MDA-AENMF deep learning model, which utilizes autoencoders and non-negative matrix factorization, efficiently predicts metabolite-disease associations and has been validated in multiple cases [50]. We look forward to combining this model with lncRNA and mRNA expression data to further explore the metabolic regulation of lncRNAs in diseases such as lung cancer, providing new strategies for disease diagnosis and treatment.

In conclusion, by integrating research achievements and technological innovations from multiple fields, we can achieve breakthroughs in the field of lung cancer treatment. We look forward to applying these cutting-edge technologies to clinical practice in future research, continuously improving the treatment effect and quality of life for lung cancer patients, and bringing greater hope to patients. At the same time, we will also focus on combining experimental validation with other computational methods to ensure the accuracy and reliability of our research results.

## Conclusion

The ceRNA mechanism, involving genes like TENM4, XRCC2, HDAC5, CDKN1A, ARFGEF3, SNAP25-AS1, RP11-58O9.2, LINC01164, VLDLR-AS1, and RP11-14I17.2, may play a role in lung adenocarcinoma (LUAD) development linked to tumor hypoxia. Quantitative polymerase chain reaction (QPCR) analysis showed decreased expression of SNAP25-AS1, RP11-58O9.2, and TENM4 in hypoxic cancer cells compared to normoxic cells. Conversely, increased expression of LINC01164, VLDLR-AS1, and CDKN1A was observed in hypoxic cancer cells.

## Supporting information

S1 Fig Dissociation curve. (TIF)

S2 Fig Amplification curve. (TIF)

S1 Table Raw data from the qRT-PCR experiments. (XLS)
