# Supplementary figures and images for: Identifying crucial lncRNAs and mRNAs in hypoxia-induced A549 lung cancer cells and investigating their underlying mechanisms via high-throughput sequencing
Source: PLoS One. 2024 Sep 5;19(9):e0307954. doi: 10.1371/journal.pone.0307954 (PMC11376552; doi:10.1371/journal.pone.0307954)

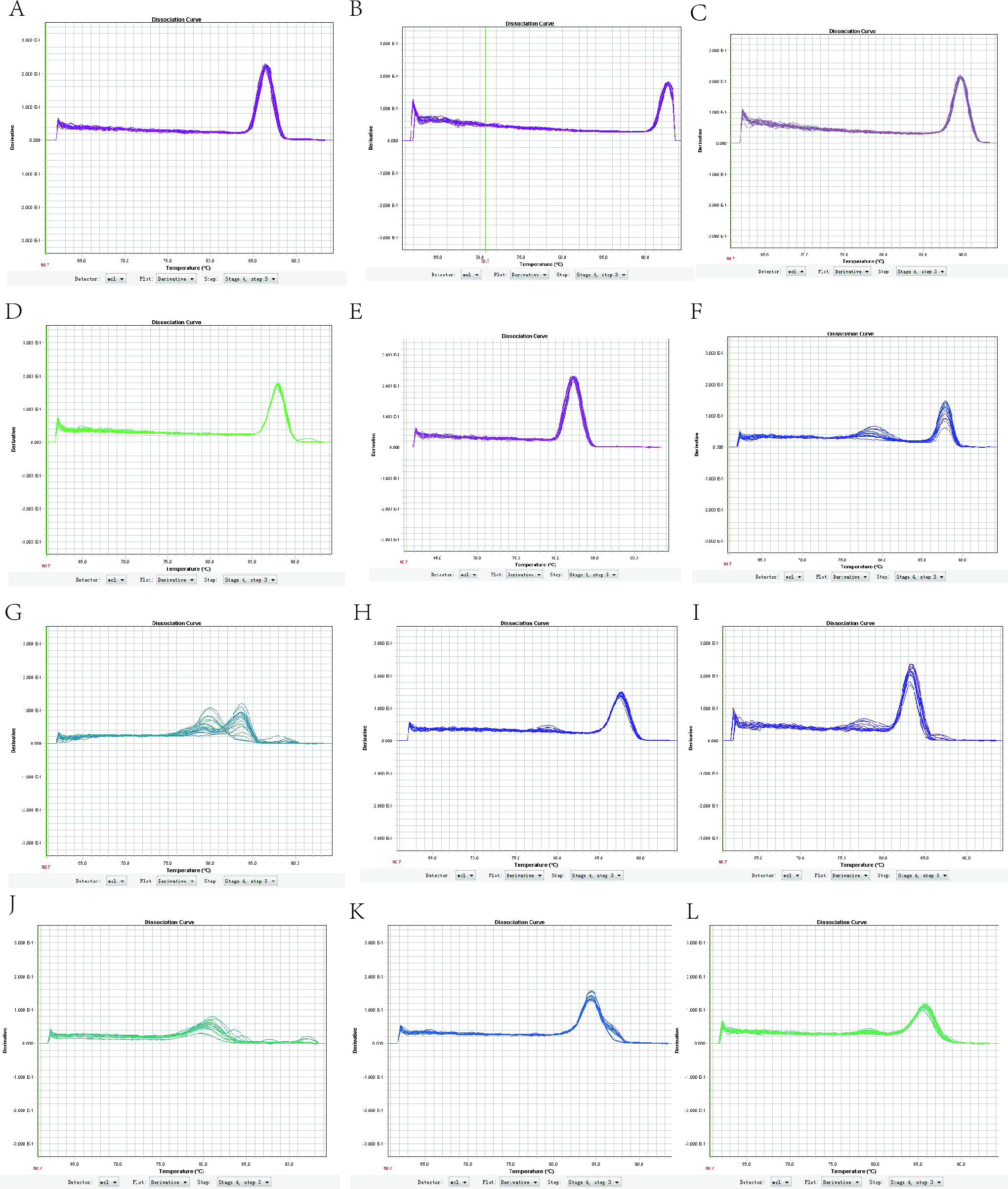

Supplement: S1 Fig — (TIF) [file pone.0307954.s001.tif]

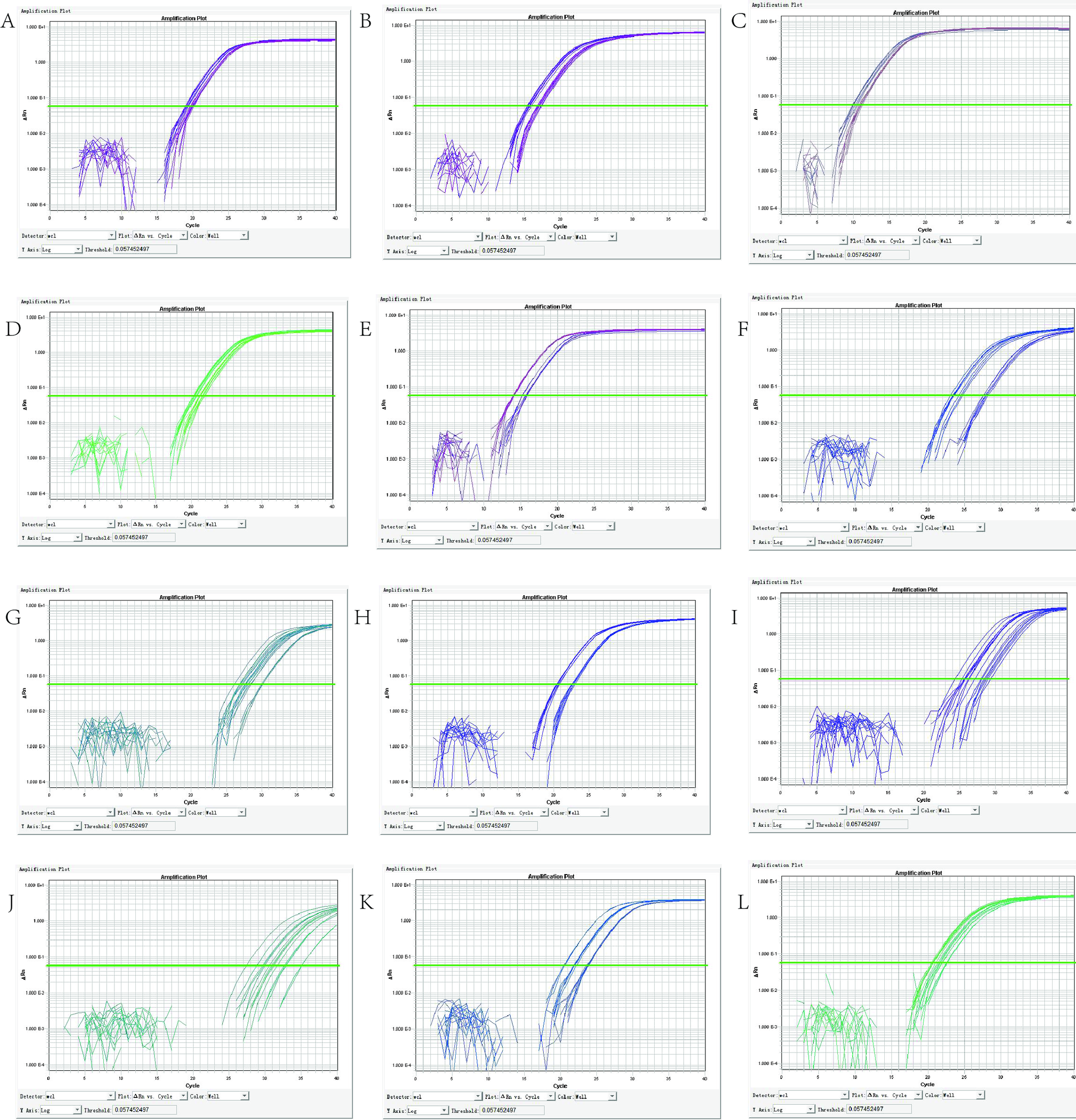

Supplement: S2 Fig — (TIF) [file pone.0307954.s002.tif]
